# Left Bundle Branch Pacing: Unexpected Resynchronization Effect of Anodal Capture

**DOI:** 10.19102/icrm.2022.130303

**Published:** 2022-03-15

**Authors:** Ronpichai Chokesuwattanaskul, Krit Jongnarangsin

**Affiliations:** ^1^Division of Cardiac Electrophysiology, University of Michigan Health Care, Ann Arbor, MI, USA; ^2^Department of Medicine, Faculty of Medicine, Cardiac Center, King Chulalongkorn Memorial Hospital, Chulalongkorn University, Thai Red Cross Society, Bangkok, Thailand; ^3^Center of Excellence in Arrhythmia Research Chulalongkorn University, Department of Medicine, Faculty of Medicine, Chulalongkorn University, Bangkok, Thailand; ^4^Department of Public Health, Bloomberg School of Public Health, Johns Hopkins University, Baltimore, MD, USA

**Keywords:** Anodal capture, left bundle branch pacing, right bundle branch

## Abstract

Anodal capture can provide a resynchronization effect on biventricular pacing. However, evidence of such an effect remains unclear in left bundle branch pacing (LBBP). We report a patient with a baseline sinus rhythm with complete atrioventricular block and a ventricular escape rhythm with a right bundle branch (RBB) block QRS morphology and left axis deviation who received LBBP for cardiac resynchronization therapy (CRT), and its anodal capture stimulation (ANS) rendered a near-normal QRS morphology. The resolution of the RBB pattern during bipolar pacing in this patient resulted from a fusion of LBBP and right ventricular septal capture from ANS. In conclusion, CRT can be achieved during LBBP by ANS in patients who do not have retrograde RBB activation.

## Introduction

Left bundle branch (LBB) pacing (LBBP) is increasingly being performed, especially in patients with high-grade degree atrioventricular block (AVB), to avoid complications related to non-physiologic ventricular depolarization from pure myocardial pacing. Anodal capture has been demonstrated to have beneficial effects on cardiac resynchronization therapy (CRT) with biventricular pacing. However, there is still a lack of data on the effect of anodal capture in LBBP.

## Case presentation

**Figure 1:**
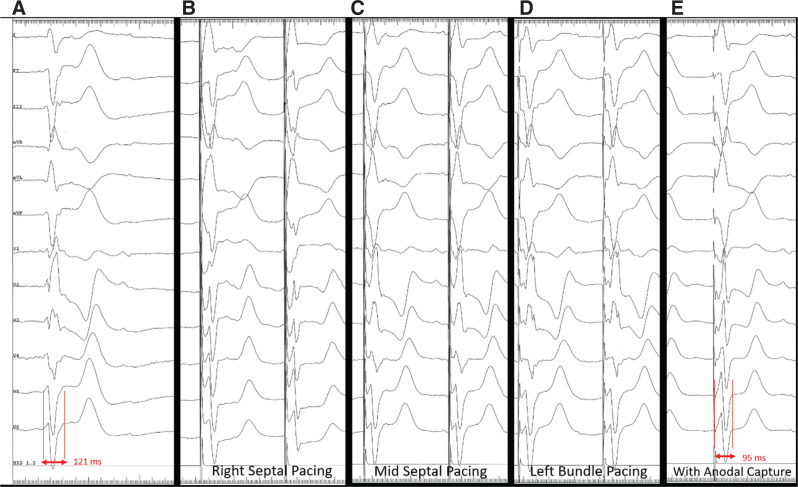
Tracings show QRS morphologies provided by different pacing sites during left bundle branch (LBB) area lead advancement. **A:** Baseline electrocardiogram demonstrates complete right bundle branch block QRS morphology for ventricular escape rhythm. **B:** “W” pattern in V1 on the initial target site for the LBB pacing (LBBP) area. **C:** Notch in V1 moving toward the end of the QRS complex as further lead advancement was performed. **D:** LBBP with a QRS morphology similar to the baseline QRS complex. **E:** High-output unipolar pacing rendered a near-normal QRS morphology as a result from a fusion of LBBP and right ventricular septal capture from anodal stimulation.

A 67-year-old woman presented for an elective upgrade from a dual-chamber pacemaker to a cardiac resynchronization therapy defibrillator (CRT-D) due to pacing-induced left ventricular (LV) systolic dysfunction. Her past medical history was significant for her complete heart block status after a dual-chamber permanent pacemaker implant, coronary artery disease status after coronary artery bypass graft surgery, and hypertension. Her baseline electrocardiogram (ECG) showed normal sinus rhythm with complete heart block. She had an escape rhythm with a right bundle branch (RBB) block morphology with a QRS duration of 121 ms and a left axis deviation (LAD) at a rate of 30 bpm **(Figure 1A)**. His-bundle lead placement was attempted but was unsuccessful due to the absence of the His-bundle potential. LBBP lead placement was then performed as described by Wu et al.^1^

## Discussion

**Figure 2:**
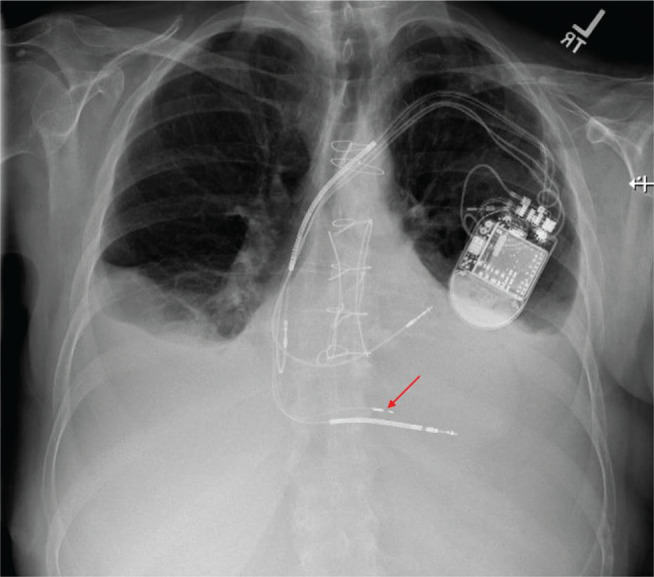
Chest X-ray anteroposterior view demonstrated the location of the left bundle branch pacing lead (arrow).

**Figure 1** shows a set of surface ECG tracings from baseline to pacing at different sites during lead advancement. Her baseline ventricular escape rhythm most likely originated either from the left posterior fascicle or LBB with underlying left anterior fascicular block given the absence of the His-bundle potential and right bundle branch (RBB) block–LAD QRS morphology. Unipolar pacing at the target site for the LBB on the right ventricular (RV) septal area demonstrated a typical “W” pattern in V1 **(Figure 1B)**. The lead was rapidly rotated clockwise until the tip penetrated the septal surface and reached the mid-septal area. The paced QRS morphology at this site demonstrated that the notch in lead V1 moved toward the end of the QRS complex **(Figure 1C)**. Further lead advancement resulted in a more prominent R-wave on the paced QRS morphology in lead V1. As the tip of the lead reached the LBB area **(Figure 2)**, the paced QRS morphology showed an RBB pattern with LAD **(Figure 1D)**, which was similar to the baseline QRS morphology indicating LBB or left posterior fascicle capture. There were no changes in QRS morphology during decremental unipolar pacing at this site, starting from 5.0 V at 0.5 ms to complete loss of capture at 0.8 V at 0.5 ms. After the delivery sheath was pulled back and the ring electrode was exposed, a repeat decremental pacing in a bipolar configuration was performed at 5.0 V at 0.5 ms. Paced QRS morphology at a high output resulted in a narrower QRS width and the resolution of the RBB pattern **(Figure 1E)**. As the pacing output was decreased to 3.0 V at 0.5 ms, the QRS morphology changed to an RBB pattern with a wider QRS width similar to the baseline QRS morphology and unipolar paced QRS morphology.

CRT with a near-normal QRS complex can be achieved during LBBP by a retrograde RBB activation in some patients. However, this patient did not have a retrograde RBB activation as demonstrated by there being no resolution of RBB block morphology during unipolar pacing. The narrow QRS morphology with a near-normal QRS morphology during bipolar pacing in this patient resulted from a fusion of LBBP and RV septal capture from anodal stimulation (ANS). ANS is defined as a capture at the anode,^2^ which is the ring electrode in this bipolar configuration. Capture usually occurs at the tip electrode (cathode) and requires a lesser pacing output than ANS. ANS can also be observed in CRT devices that allow pacing from the LV lead electrode as a cathode to the RV lead ring or coil as an anode. ANS from the RV lead electrode can result in biventricular capture during LV pacing alone or triple-site ventricular capture during biventricular pacing.^3,4^ In summary, CRT can be achieved during LBBP by ANS in patients who do not have retrograde RBB activation. However, the benefit of CRT from ANS in LBBP is unclear and requires more clinical studies.
